# Supplementary material for: Metabolic Syndrome and Its Associated Early-Life Factors among Chinese and Spanish Adolescents: A Pilot Study
Source: Nutrients. 2019 Jul 11;11(7):1568. doi: 10.3390/nu11071568 (PMC6682950; doi:10.3390/nu11071568)
Supplement: Supplementary file 1 [file nutrients-11-01568-s001.pdf]

Supplementary Table S1. Risk of MetS features based on associated factors from multivariable logistic regression (categorical variables).

**Categorical Variables (reference)**	**N**	**MetS**
		OR(95%CI)
Age		1.01 (0.80,1.29)
Gender (Boy)	1011	1.48 (0.69,3.19)
Country (China)	1150	**14.52 (5.53,38.13)**
Breastfeeding duration (≤6 month)	732	**0.50 (0.20,0.89)**
Birth weight (normal weight)	1720	
High birth weight	193	1.24 (0.46,3.36)
Low birth weight	89	NA
